# Collagen targeted self-anchoring antimicrobial molecular agents

**DOI:** 10.3389/fbioe.2026.1914224

**Published:** 2026-10-02

**Authors:** Nur Ceren Selcuk, Erhan Demirel, Stephen C. Waller, Malcolm L. Snead, Paulette Spencer, Candan Tamerler

**Affiliations:** 1 Bioengineering Program, University of Kansas, Lawrence, KS, United States; 2 Institute for Bioengineering Research, University of Kansas, Lawrence, KS, United States; 3 Department of Internal Medicine, Division of Infectious Diseases, University of Kansas Medical Center, Kansas City, KS, United States; 4 Center for Craniofacial Molecular Biology, Herman Ostrow School of Dentistry of USC, The University of Southern California, Los Angeles, CA, United States; 5 Department of Mechanical Engineering, University of Kansas, Lawrence, KS, United States

**Keywords:** antimicrobial peptides, collagen targeting, infection, molecular therapeutic agents, multifunctional, self anchoring

## Abstract

The intrinsic characteristics of collagen, when combined with its natural abundance in the tissues and its easy exposure due to any fissure of epithelium during a wound or an injury, makes it a common site for bacterial colonization. Within dense collagen fibrils, such as those in dentin, bound pathogens are partially shielded from antibiotic penetration. With the increasing prevalence of antibiotic resistance, microbial infections remain a persistent global health concern. In this study, we engineered a collagen targeted self-anchoring antimicrobial peptide as a novel therapeutic agent and tested its multifunctional activities on a collagen platform. Our peptide design incorporated an antimicrobial peptide (AMP) with another peptide designed for collagen-binding properties through a spacer domain. The novel collagen targeted AMP (COLTAMP) peptide was computationally evaluated for its potential to simultaneously exhibit both desired functions, collagen-binding and antibacterial activity. The predicted secondary structure of COLTAMP was confirmed using Fourier-transform infrared and Raman spectroscopies as complementary vibrational techniques. The interactions of the targeted AMP with collagen fibrils were characterized using atomic force microscopy, providing insights into the effects of the peptide on the organization of fibrils. The antimicrobial activity of the targeted AMP was tested using a zone of inhibition assay against a model pathogen, *Streptococcus* mutans, which thrives in the oral cavity and colonizes distant tissues once it enters the bloodstream. Confirming the antimicrobial activity of COLTAMP, we next explored its mechanism of action using a scanning electron microscopy (SEM). SEM imaging confirmed bacterial cell wall disruption following the delivery of COLTAMP, leading to pronounced effects on bacterial membrane integrity. The proposed self-anchoring targeted multifunctional peptide offers spatially distributed antimicrobial peptides across collagen fibrils. Multifunctional targeted peptide agents that selectively anchor bioactive domains directly to damaged collagen offer potential treatment strategies for infections involving diverse injury sites.

## 1 Introduction

Microbial infections remain a pressing global health concern, exacerbated by the increasing prevalence of antimicrobial resistance (AMR) (Voidarou et al., 2011; Vera et al., 2012; Tan and Tatsumura, 2015; Cai and Liu, 2020; Nelli et al., 2022). In the United States, the Centers for Disease Control and Prevention (CDC) reports over 2.8 million cases of AMR-related infections annually, resulting in more than 35,000 deaths (Solomon and Oliver, 2014; Suetens et al., 2018). The economic burden of AMR is equally alarming, with bacterial resistance alone contributing over 20 billion dollars in additional healthcare costs annually in the United States and exceeding nine billion dollars in Europe (Prestinaci et al., 2015; Dadgostar, 2019). Traditional approaches, such as antibiotics, although initially effective, have been undermined by the rapid emergence of drug-resistant bacterial strains, leading to limited efficacy and unintended consequences, including longer hospital stays and higher treatment costs (Solomon and Oliver, 2014; Dadgostar, 2019; Majumder et al., 2020; Murugaiyan et al., 2022). These challenges necessitate the development of alternative strategies for infection control that are sustainable, effective, and adaptable to various clinical scenarios. Among these, peptide-based delivery systems combined with antimicrobial agents have gained considerable attention. Collagen scaffolds have emerged as key candidates for the direct integration of antimicrobial agents.

Collagen, a major structural component of the extracellular matrix (ECM), mimics the physical architecture of tissues and serves as a versatile scaffold owing to its excellent biocompatibility, biodegradability, and ability to support cellular attachment and growth (Ricard-Blum, 2011; Sorushanova et al., 2019). These intrinsic characteristics, combined with its natural abundance and adaptability for functionalization, make collagen an ideal biomaterial candidate for the development of bioactive scaffolds for tissue engineering and regenerative medicine (Lee et al., 2001; Singh et al., 2011; Sundar et al., 2021). Collagen scaffolds are widely used to facilitate tissue reconstruction for bone and tendon repair. The clinical uses of collagen ECMs in tissue reconstruction and repair are broad. Such uses include tissue repair in the head and neck, oral cavity, bone fracture repair, and tendon repair. Many of the applications for collagen scaffolds occur in or adjacent to non-sterile environments and thus, the provision of a scaffold that inhibits microbial colonization is key to good clinical outcomes. The functionalization of collagen scaffolds offers a tremendous opportunity for the direct integration of novel therapeutics to provide localized treatments (Rezvani Ghomi et al., 2021; Ersanli et al., 2023; Fan et al., 2023).

The inherent susceptibility of collagen to microbial colonization and biofilm formation, particularly in wound care, bone defects, and dental applications, underscores the need to enhance its antimicrobial activity. Bacterial attachment is the first step in establishing bacterial colonization. Collagen binding proteins allow for ready bacterial adhesion to host cells, followed quickly by biofilm formation. The presence of biofilms offers structural interference with immune cell phagocytosis of colonized bacteria. With biofilm maturation, persister cells, a subpopulation of slow growing bacteria with decreased susceptibility to antimicrobials, quickly dominate the biofilm (Percival et al., 2015; Fisher et al., 2017; Miyaue et al., 2018; Arora et al., 2021; Zhao et al., 2023; Xu et al., 2024). Antimicrobial peptides (AMPs) can function effectively in biofilm environments unlike most antibiotics, AMPs do not rely on cell division and active cellular metabolism to be effective (Zhang et al., 2021; Xuan et al., 2023).

AMPs have received increasing attention as promising therapeutic candidates because of their pathogen-killing ability and low or no antibiotic resistance (Wang et al., 2019; Moretta et al., 2021; Dini et al., 2022). They hold promises for addressing the challenge of emerging antibiotic resistance and can be optimized for enhanced efficacy through peptide engineering (Yucesoy et al., 2015; Yazici et al., 2016; Ye et al., 2017; Boone et al., 2018; Xie et al., 2019; Wisdom et al., 2020; Yuca et al., 2021; Boone et al., 2024). These peptides serve as integral components of innate immune defense in all forms of life. AMPs are typically composed of short amino acid sequences ranging from five to fifty residues. AMPs exert antimicrobial effects through their unique structure and physicochemical properties, including conformation, charge distribution, hydrophilicity, and hydrophobicity (Luo and Song, 2021; Talapko et al., 2022). Their amphipathic nature enables interactions with negatively charged microbial cell membranes, disrupting their integrity and ultimately causing cell death. Numerous studies have demonstrated the potential of AMPs to enhance the antimicrobial properties of biomaterials, making them valuable tools in the development of infection-resistant medical applications (Yucesoy et al., 2015; Cassin et al., 2016; Wisdom et al., 2016; Yazici et al., 2019; Wisdom et al., 2020; Xie et al., 2020; Ye et al., 2021; Ye et al., 2022).

Engineering delivery systems with antimicrobial peptides (AMPs) and collagen-binding peptides (CBPs) offer unique opportunities for directly integrating antimicrobial agents into collagen scaffolds and damaged tissues. CBPs specifically bind to collagen fibrils, stabilize their structure, and mitigate enzymatic degradation (Wahyudi et al., 2016; Boone et al., 2023; Cloyd et al., 2023). Targeting collagen by selectively anchoring AMPs holds promise for diverse applications, including wound healing, infection prevention in tendons, bone tissue engineering, and dental restorations (Ersanli et al., 2023).

In this study, we designed a novel multifunctional peptide that combines an AMP domain with a collagen-targeted self-anchoring domain as an antimicrobial therapeutic agent. This multifunctional peptide was evaluated within a biomimetic platform mimicking a collagen fibril, which allowed us to investigate its efficacy in stabilizing collagen and its ability to inhibit microbial colonization, as well as its mechanism of action. Recognizing the diverse applications of collagen-antimicrobial biomaterials, we selected *Streptococcus mutans* (*S. mutans*) as a model organism to assess peptide performance. This pioneer cariogenic bacterium is a key contributor to dental caries and the failure of dental composite restorations through the infiltration of exposed collagen fibrils at the dentin interface (Jaffer et al., 2002; Seneviratne et al., 2011; Bourbia et al., 2013; Ionescu et al., 2015; Bächle et al., 2023; Mokeem et al., 2023). This bacterium was chosen not only for its role in dental caries and composite restoration failure but also as an example of the microbial challenges faced in collagen-rich environments. Our study provides details on the engineered collagen-targeted antimicrobial peptide (COLTAMP) and offer insights into its mechanism of action using a collagen testing model. The research outcome contributes to the development of advanced collagen-antimicrobial biomaterials with peptide-enabled tunable properties, addressing critical challenges in infection control.

## 2 Materials and methods

### 2.1 Materials

*N,N*-Dimethylformamide (DMF, 99.8%), dichloromethane (DCM, 99.8%), trifluoroacetic acid (TFA, 99%), triisopropylsilane (98%), thioanisole (99%), diethyl ether (99%), collagen type 1 solution from rat tail (3 mg/mL, 95%), chlorhexidine digluconate (CHX, 20% in aq. soln.) were obtained from Sigma-Aldrich Inc. (St. Louis, MO, United States). Rink resin, Fmoc-resin, Fmoc-amino acid building blocks, piperidine, and 2-(1H-benzotriazole-1-yl)-1,1,3,3-tetramethyluronium hexafluorophosphate (HBTU) were purchased from AAPPTec LLC (Louisville, KY, United States). 1,2-ethanedithiol (95%), *N*-methyl morpholine (NMM, 99%), glutaraldehyde (25% aq. soln.), Bacto™ brain heart infusion (BHI), BD BBL dehydrated brain heart infusion agar, and Corning™ Clear polystyrene 96-well microplates (Corning, NY, United States), were obtained from Thermo Fisher Scientific (Vilnius, Lithuania). *Streptococcus mutans* UA159 was obtained from the American Type Culture Collection (ATCC® 25175™; Manassas, VA, United States). Highest grade V1 12 mm mica discs and 12 mm atomic force microscopy specimen discs were obtained from Ted Pella Inc. (Redding, CA, United States). STKYDOT, an atomic force microscopy (AFM) specimen disc adhesive, was purchased from Bruker Corporation (Camarillo, CA, United States).

### 2.2 Multifunctional peptide design

We selected AMP2 (KWKRWWWWR) as an antimicrobial domain for the multifunctional design. It is a computationally designed and characterized antimicrobial peptide that has been generated by generic algorithms (GA) and machine learning algorithms (Fjell et al., 2011), and we have selected this peptide because of its high activity at low concentrations against *S. mutans* (Yuca et al., 2021). LRR-10 (LRELHLNNN) is a decorin subunit that demonstrates a high affinity and stability for type I collagen (Federico et al., 2015). We employed a -GGG- spacer between the two active domains to provide distance and flexibility to both domains. The physicochemical properties of the peptides were calculated using the Expasy Protparam (Gasteiger et al., 2005) and Pep-Calc.com (Lear and Cobb, 2016) peptide property calculation tools. Theoretical folding and secondary structures were obtained using PEP-FOLD 3.5 (Lamiable et al., 2016) and further processed using ChimeraX 1.8 software (Pettersen et al., 2021).

### 2.3 Peptide synthesis

Peptides were synthesized using an automated solid-phase peptide synthesizer (AAPPTEC Focus XC, Louisville, KY, United States) and standard Fmoc chemistry protocol. The peptides were synthesized on Rink resin, and the crude resins were cleaved from the resin and side-chain deprotected using Reagent K (TFA/triisopropylsilane/H_2_O/ethanedithiol/thioanisole/phenol (80:1:5:2.5:5:5) and precipitated in cold ether. The peptide was lyophilized and stored at −20 °C. Peptide solutions were freshly prepared from stock powders for each experiment prior to use. The molecular weights and purities of the peptides were confirmed using mass spectroscopy.

### 2.4 Fourier transform-infrared spectroscopy (FTIR)

FTIR spectroscopy was performed using a PerkinElmer Frontier IR spectrometer equipped with an attenuated total reflectance (ATR) accessory (Waltham, MA, United States). Spectra were acquired over the range of 650–4,000 cm^−1^ at a spectral resolution of 4 cm^−1^, with 20 acquisitions per cycle averaged to improve the signal-to-noise ratio. The lyophilized peptide powder was placed directly onto the ATR crystal in sufficient quantity to ensure complete coverage of the crystal surface, and consistent contact pressure was applied using the instrument’s pressure arm. The collected spectra were analyzed to characterize the secondary structure of the peptide by examining the amide A, amide I, and amide II band positions and the chemical composition of its constituent amino acid residues, including the identification of characteristic vibrational modes associated with the indole side chains of tryptophan residues and skeletal C-N and C-C stretching features of the peptide backbone. Spectral analysis and quantification were carried out using Spectragryph Version 1.2.16.1 optical spectroscopy software.

### 2.5 Raman spectroscopy

Raman spectroscopy was performed using a LabRAM ARAMIS Raman microscope (HORIBA Jobin Yvon, Edison, NJ, United States) equipped with a 785 nm diode laser (LD current: 200 mA; Sacher Lasertechnik PilotPC 500, Marburg, Germany) and a motorized stage (Märzhäuser Wetzlar GmbH & Co. KG, Wetzlar, Germany). The lyophilized peptide powder was deposited onto clean glass slides, which were wrapped in aluminum foil to minimize background fluorescence and improve the signal-to-noise ratio. Spectra were collected over the wavenumber range of 200–1800 cm^−1^ using a ×100 objective, 1,200 gr/mm grating, 500 μm confocal hole, and 300 μm entrance slit, with an exposure time of 24 s and 16 acquisitions per cycle. In addition to secondary structure assessment via the amide I and amide III band regions, the spectra were analyzed for residue-specific vibrational features, with particular attention to the characteristic indole ring breathing modes, Fermi resonance doublets, and N-H bending vibrations of the four tryptophan residues, as well as the contributions from arginine, histidine, and the peptide backbone. All spectral data were processed and analyzed using the LabSPEC 6 software (HORIBA Jobin Yvon, Edison, NJ, United States).

### 2.6 Bacterial maintenance and culturing

Antimicrobial activity assays were conducted using the *Streptococcus* mutans UA159 bacterial strain (ATCC® 25175™). The ATCC standard protocol was followed to prepare the *S. mutans* culture, where the lyophilized bacteria were inoculated in Brain Heart Infusion (BHI) media, and the stock culture plate was prepared by streaking onto prepared BHI agar, followed by incubation overnight at 37 °C in a 5% carbon dioxide (CO_2_) atmosphere. Prior to each experiment, *S. mutans* UA159 overnight cultures were freshly prepared from a single colony-forming unit, inoculated in 10 mL of fresh BHI medium, and incubated for 14 h at 37 °C in a 5% CO_2_ atmosphere until the mid-logarithmic phase. Bacterial growth was monitored by measuring the optical density at 600 nm (OD600) using a Cytation 3 imaging multimode plate reader (BioTek Instruments, Inc., Winooski, VT, United States).

### 2.7 Zone of inhibition tests

*The S. mutans* bacterial strain was grown according to the protocol discussed in the Bacterial Maintenance and Culturing section. Overnight *S. mutans* cultures at the mid-logarithmic phase were spread onto BHI agar plates using sterile cotton swabs. Empty discs were placed on agar plates in triplicate, and 10 μL of the peptide dissolved in deionized water was pipetted onto each disc. A 63 μg/mL CHX solution was used as the positive control, and deionized water served as the negative control. The plates were incubated for 24 h at 37 °C in the presence of 5% CO_2_, and the zone of inhibition of bacterial growth was measured and photographed using a smartphone camera equipped with a macro lens attachment. Images were analyzed using ImageJ Fiji v1.54 (Schneider et al., 2012) to measure the inhibition zones. The mean and standard deviation of the zone of inhibition were calculated.

### 2.8 Antimicrobial activity assay

Inhibitory concentration assays were conducted using a standard broth microdilution method according to the Clinical and Laboratory Standards Institute (CLSI) protocol (Ferraro, 2000; Ye et al., 2009; Parthasarathy et al., 2012; Min et al., 2017; Moussa et al., 2019; Yuca et al., 2021). To conduct the in solution antimicrobial activity assay, two-fold serial dilutions in deionized water were prepared in 96-well plates to achieve final concentrations of peptide ranging from 1 to 64 μg/mL in a volume of 20 μL. This step was followed by the addition of 80 μL of BHI broth and 100 μL of the final bacterial culture containing 1 × 10^4^ CFU/mL. Three different controls were employed for the assay. The wells containing only deionized water without any antimicrobial agent served as a negative control to monitor bacterial growth. The positive control for antibacterial activity contained 20 μL of 63 μg/mL CHX in deionized water. The positive control was used to monitor full bacterial growth inhibition, while media and deionized water served as blank control to monitor any possible contamination of the assay. The plate was incubated overnight at 37 °C in presence of 5% CO_2_. After the incubation, 5 μL solution from each well was transferred to a new black-walled 96-well plate with 195 μL fresh BHI media. The bacterial cell viability was assessed by using Resazurin salt (Sigma-Aldrich 199303). Following overnight incubation, bacterial cultures were transferred to a sterile 96-well plate containing fresh BHI medium, and 1X resazurin solution was added at 10% (v/v) (Vieira-da-Silva and Castanho, 2023). After 2 h incubation, fluorescence was measured at Ex/Em = 565/595 nm. Minimum Inhibitory Concentrations (MIC) determined by the minimum concentration where full bacterial growth inhibition was observed.

### 2.9 Scanning electron microscopy (SEM) imaging

Bacteria grown overnight in the mid-logarithmic phase (100 μL) were treated with 100 μL of 4× MIC of AMP and COLTAMP overnight in the presence of 5% CO_2_ at 37 °C. A bacterial culture without peptide was used as a control. The peptide-treated bacteria were centrifuged at 6,000 rpm for 10 min at room temperature and washed with PBS three times. The samples were then fixed with 1 mL of 2.5% glutaraldehyde overnight, followed by washing with PBS three times. Bacteria samples were gradually dehydrated by incubating them in an ethanol series of 30%, 50%, 70%, 90% once and 100% twice, each for 10 min. After the final ethanol wash, 20 μL of ethanol was used, and the samples were deposited on positively charged glass slides. Air-dried samples were mounted on SEM stubs using double-sided carbon tape and sputter-coated with gold at a thickness of 10 nm using a Cressington Sputter Coater 108 Auto (Cressington Scientific Instruments, Inc., Watford, United Kingdom). Images were captured using a Phenom Pro X Desktop Scanning Electron Microscope (SEM) at an acceleration voltage of 15 kV with magnification settings of 30, 000× and 70, 000×.

### 2.10 Peptide incorporation into collagen as a testing model

Grade V1 mica discs, measuring 0.21 mm in thickness and 12 mm in diameter, served as the substrate surface and were affixed to a 12 mm AFM specimen disc using STKYDOT adhesive. A suspension containing 1 mL of type 1 rat tail collagen at a concentration of 3 mg/mL was introduced with 50 µmol of the peptide. After dissolving the peptide in the collagen suspension, 150 μL of the mixture was drop-casted onto the substrate surface and allowed to self-assemble at 4 °C for 16 h, resulting in a dry and thin layer of collagen-peptide coating on the substrate surface.

### 2.11 Atomic force microscopy

Topological changes on the collagen self-assembly platform before and after peptide incorporation were examined using a Bruker Multimode 8 HR scanning probe microscope (Bruker Nano Inc., Camarillo, CA, United States) in tapping mode using PeakForce in air mode at 24 °C ± 2 °C and 40% ± 5% RH conditions. Images were acquired in tapping mode using etched silicon probes (type RTESPA 525, Bruker Nano Inc., Camarillo, CA, United States) with a resonant frequency of approximately 518 kHz. 5 μm × 5 μm images were acquired at a scan rate of 0.5 Hz with a resolution of 512 pixels/line. Images of the samples were recorded using NanoScope 8.15 software and analyzed using NanoScope Analysis Software (version 3.0).

## 3 Results

### 3.1 Peptide structure and properties

The COLTAMP is engineered through a spacer design linking the collagen-binding domain and the antimicrobial domain, making it a multifunctional agent. The antimicrobial domain targets pathogens, whereas the collagen-binding domain provides structural features to the collagen fibrils. It is designed to serve a dual role in enhancing the antimicrobial properties of collagen-based scaffolds to address microbial resistance. The structural properties of COLTAMP are shown in Figure 1, its helical wheel projection is shown in Figure 2, and the physicochemical properties of the COLTAMP are summarized in Table 1.

**FIGURE 1:**
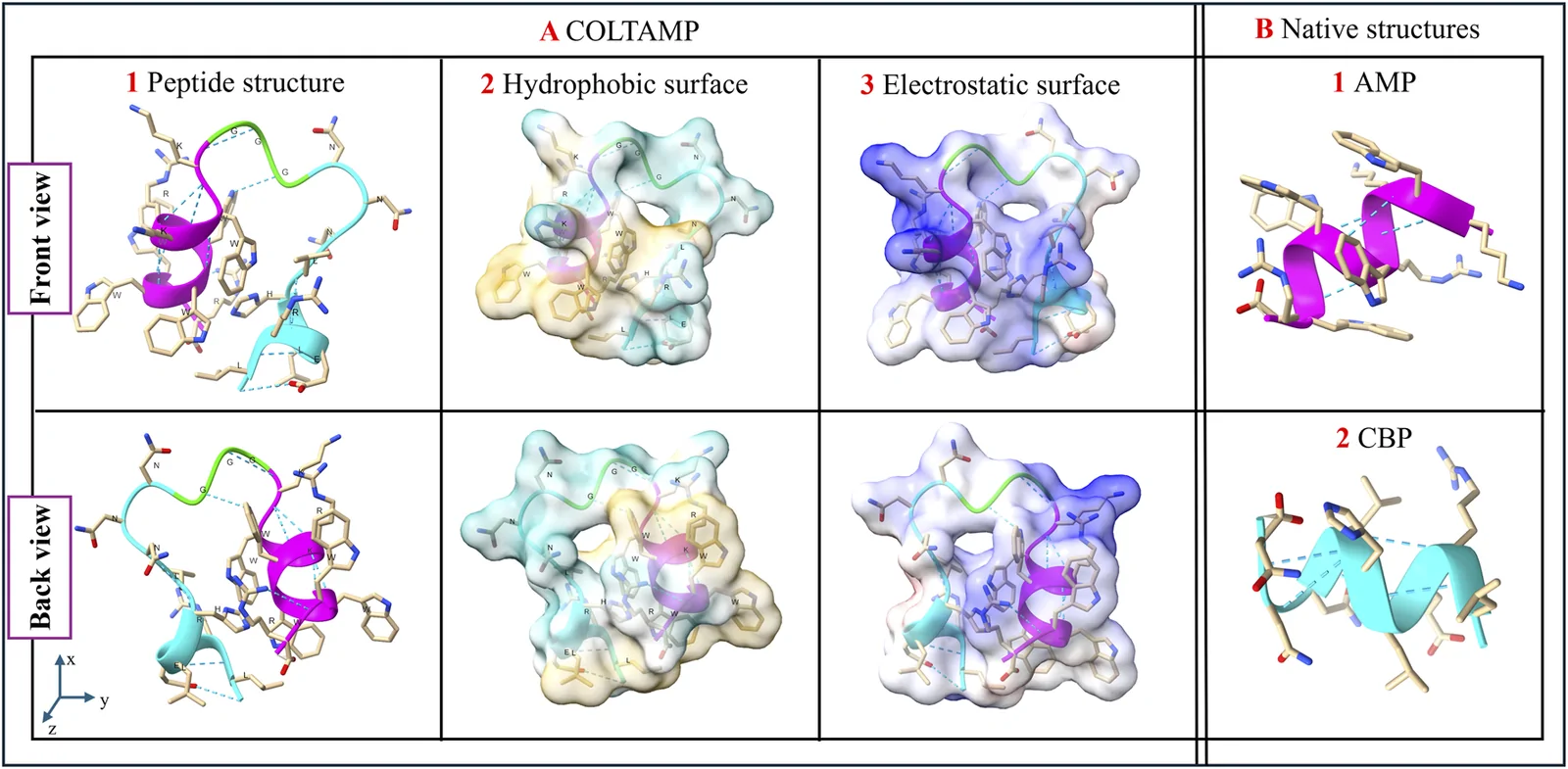
Predicted peptide structure by PEP-FOLD 3.5. **(A‐1)** Representation of COLTAMP’s 3D folding in an aqueous environment from different angles. Magenta color is the antimicrobial domain; cyan color is the collagen binding domain, and green color is the spacer. **(A‐2)** Hydrophobic surface of COLTAMP from different angles. The dark cyan represents hydrophilic surfaces, whereas the dark goldenrod represents hydrophobic surfaces. **(A‐3)** Electrostatic potential surface of COLTAMP from different angles. Red color represents negative potential through white to blue for positive potential. **(B‐1)** Representation of the AMP’s native 3D folding in aqueous environment. **(B‐2)** Representation of the CBP’s native 3D folding in aqueous environment.

**FIGURE 2:**
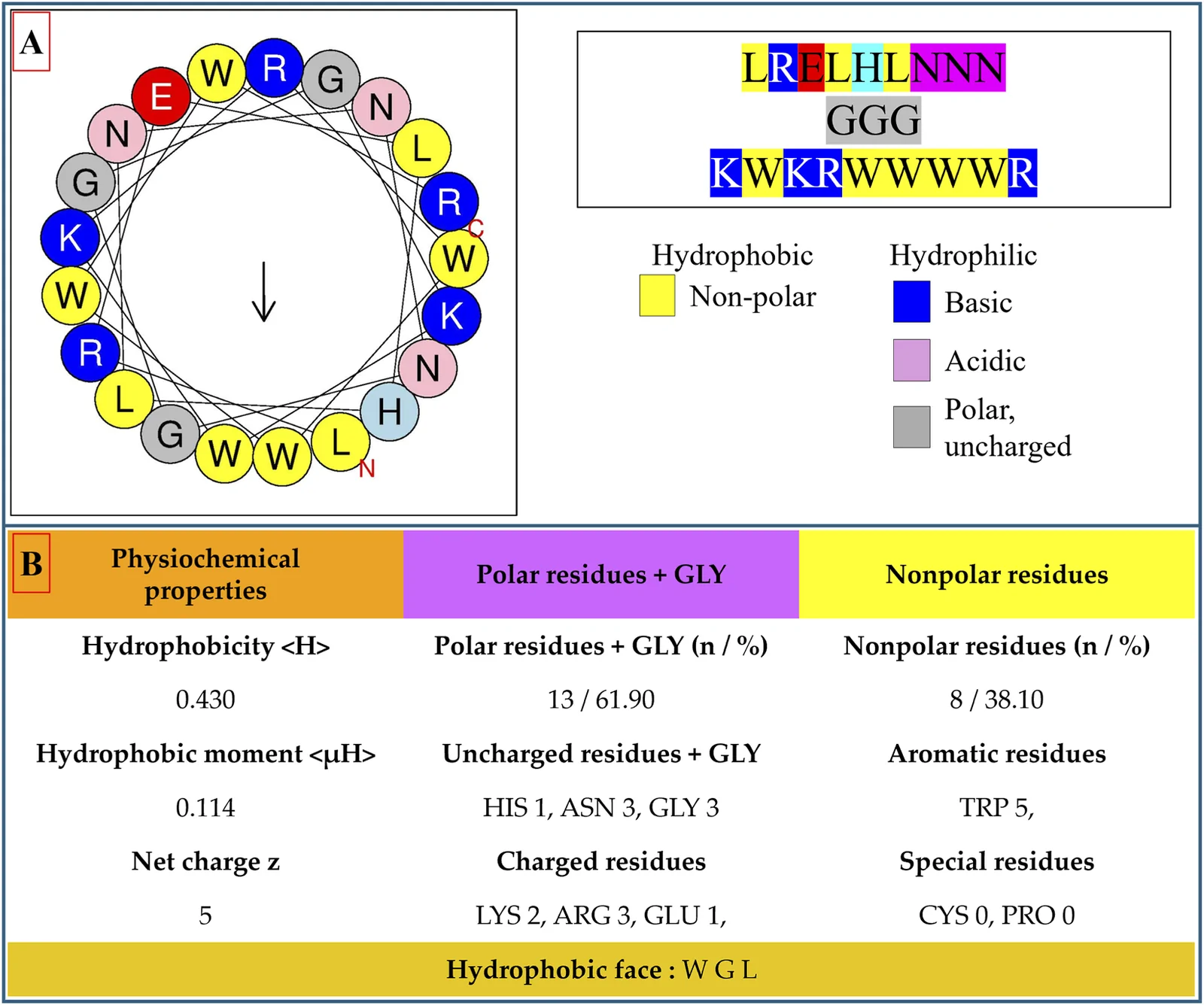
Helical wheel projection of the COLTAMP **(A)** and related properties **(B)**.

**TABLE 1:** Theoretical physicochemical properties of peptides calculated by using expasy-protparam and Pep-Calc tools.

Peptide	Peptide sequence	Molecular weight (g/mol)	Theoretical pI	Net charge at pH 7	GRAVY score
AMP	KWKRWWWWR-NH_2_	1,515.82	14.00	5	−2.367
CBP	LRELHLNNN	1,122.24	7.81	0	−1.144
COLTAMP	LRELHLNNN-GGG-KWKRWWWWR-NH_2_	2,792.17	12.43	5	−1.562

The secondary structures of COLTAMP, AMP, and CBP were predicted using PEP-FOLD 3.5 and visualized using ChimeraX 1.8 software. As shown in Figure 1‐A1, the collagen-binding domain (cyan) adopts an α-helical structure, similar to its native structure, which is consistent with its design to interact with the triple-helical structure of collagen fibrils. The α-helical configuration provides structural stability and increases the likelihood of hydrogen bonding and hydrophobic interactions with collagen. The antimicrobial domain (magenta) exhibits an α-helix and utilizes its amphipathic helices to insert into and disrupt bacterial cell membranes. This helix provides a balance between rigidity and flexibility, allowing the peptide to adapt to various surfaces. The spacer (green) allows functional domains to partially preserve their secondary structures (Figures 1B1,2) while providing separation between the domains and flexibility to the peptide, allowing the peptide to adapt its conformation to engage with both bacterial and collagen surfaces simultaneously.

The hydrophobic properties of the COLTAMP were further analyzed to better understand its functionality (Figure 1‐A2). The visualization of the hydrophobic surface revealed a hydrophobicity gradient between the domains. The collagen-binding domain displayed a more hydrophilic surface, which could contribute to its interaction with the exposed hydrophobic regions of collagen fibrils. In contrast, the antimicrobial domain exhibits a higher degree of hydrophobicity, which is critical for targeting bacterial membranes. The amphipathic character with distinct hydrophobic and hydrophilic surfaces allows the peptide to efficiently alternate between interactions with hydrophilic collagen surfaces and hydrophobic bacterial membranes, suggesting optimal dual functions in physiological environments.

The electrostatic surface representation in Figure 1‐A3 demonstrates that COLTAMP has predominantly cationic surface properties. Positive regions were concentrated in the antimicrobial domain, indicating that the AMP domain likely provides the main electrostatic driving force for interactions with negatively charged targets, such as bacterial cell walls. In contrast, the negative potential was limited to small and localized surface patches, whereas the remaining regions were largely neutral to moderately polar. This distribution indicates that the peptide does not possess a random surface charge pattern but instead displays an asymmetric electrostatic organization consistent with a functional cationic peptide architecture.

Taken together, the structural model, hydrophobic surface map, and electrostatic surface map indicate that COLTAMP adopts distinct but coordinated surface properties. When compared, the hydrophobic and electrostatic maps suggest a partially amphipathic surface arrangement, where cationic regions likely support the initial electrostatic recognition of negatively charged surfaces, and hydrophobic regions may contribute to stabilization after binding. Simultaneously, the collagen-binding domain remains structurally exposed, supporting the idea that COLTAMP preserves the accessibility of both functional domains. Overall, these results suggest that the peptide maintains a balanced surface organization compatible with dual functionality, combining antimicrobial surface recognition with collagen-targeting capability.

The helical wheel projection of COLTAMP (Figure 2) was generated using HeliQuest (Gautier et al., 2008), further illustrating the amphipathic properties of the α-helices within the peptide. The helical wheel plot revealed a dichotomy between the hydrophobic and hydrophilic faces of the peptide. Residues such as leucine, tryptophan, and glycine cluster on the hydrophobic face, forming a surface ideal for interaction with lipid membranes. In contrast, the hydrophilic face is populated with charged residues, such as lysine, arginine, and glutamic acid. These positively charged amino acids interact with the negatively charged groups present in collagen fibrils. This arrangement enhances electrostatic interactions, suggesting that the peptide can bind to collagen surfaces to promote the structural integrity of collagen-based scaffolds. The helical wheel projection highlights the peptide’s design as an amphipathic helix, capable of reorienting itself based on the surrounding environment.

The theoretical physicochemical properties of the peptides (Table 1) were calculated using the Expasy Protparam (Gasteiger et al., 2005) and Pep-Calc.com (Lear and Cobb, 2016) peptide property calculator tools. The C-termini of both AMP and COLTAMP were amidated during synthesis to improve their antimicrobial properties. The AMP peptide has the highest theoretical pI (14) and a positive net charge of +5 at pH 7, reflecting its high content of positively charged residues such as lysine (K) and arginine (R). A GRAVY score of −2.367 indicates hydrophilicity, attributed to its amphipathic nature and presence of tryptophan (W) residues, which also contribute to its aromatic and hydrophobic character. CBP is smaller, with a molecular weight of 1,122.24 g/mol. Its theoretical pI (7.81) is close to neutral, resulting in a net charge of approximately 0 at pH 7. The GRAVY score of −1.144 indicates good hydrophilicity, providing the peptide with good solubility in water. The COLTAMP, which combines the CBP and AMP sequences through a flexible glycine spacer (GGG), has the highest molecular weight (2,792.17 g/mol) and a theoretical pI of 12.43. At pH 7, it carries a positive net charge of +5, similar to the AMP domain, and a GRAVY score of −1.562, indicating good hydrophilicity and solubility.

Overall, physicochemical analysis highlights the deliberate design of the COLTAMP, which integrates the antimicrobial efficacy of AMP with the collagen targeting properties of CBP.

### 3.2 FTIR and Raman spectroscopy analysis of COLTAMP

The predicted secondary structure was confirmed using FTIR and Raman spectroscopy. FTIR spectroscopy was used to identify the amino acid signatures and the characteristic secondary structure of the peptide (Figure 3), and the findings were correlated by analyzing the peptide using Raman spectroscopy (Figure 4).

**FIGURE 3:**
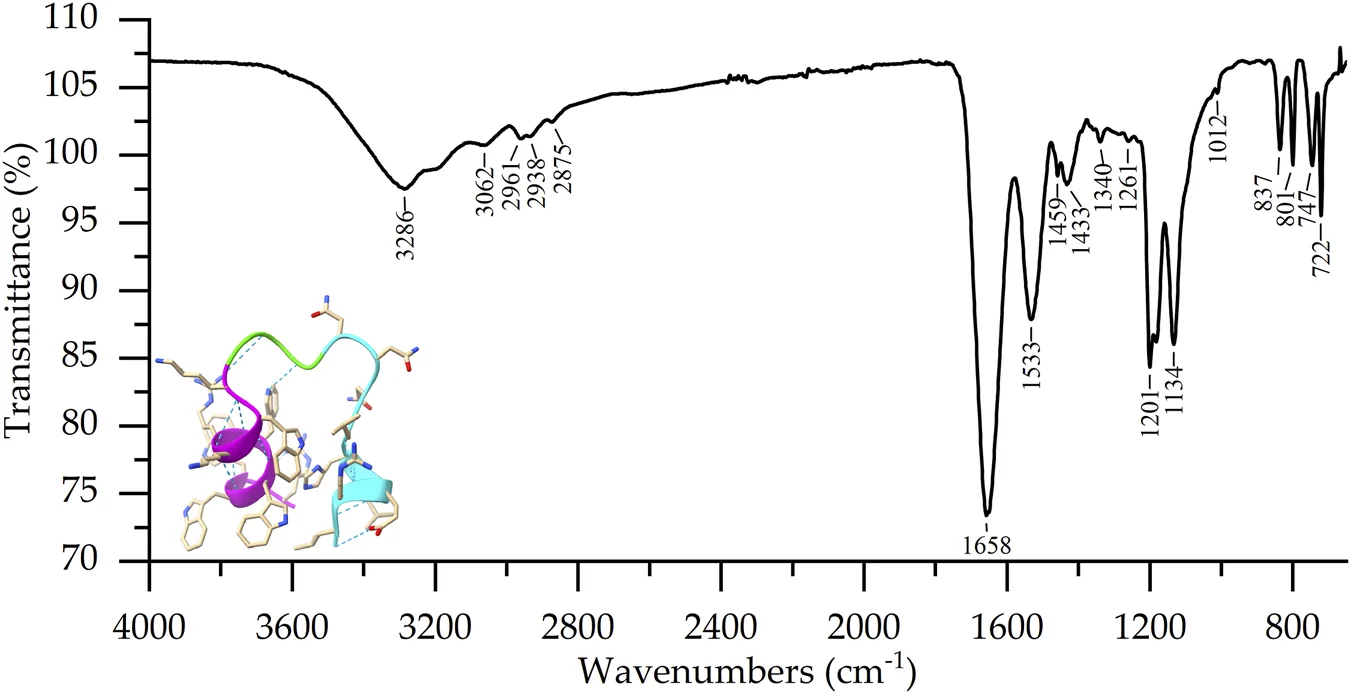
ATR-FTIR spectrum of the COLTAMP. Inset provides the predicted multifunctional peptide structure.

**FIGURE 4:**
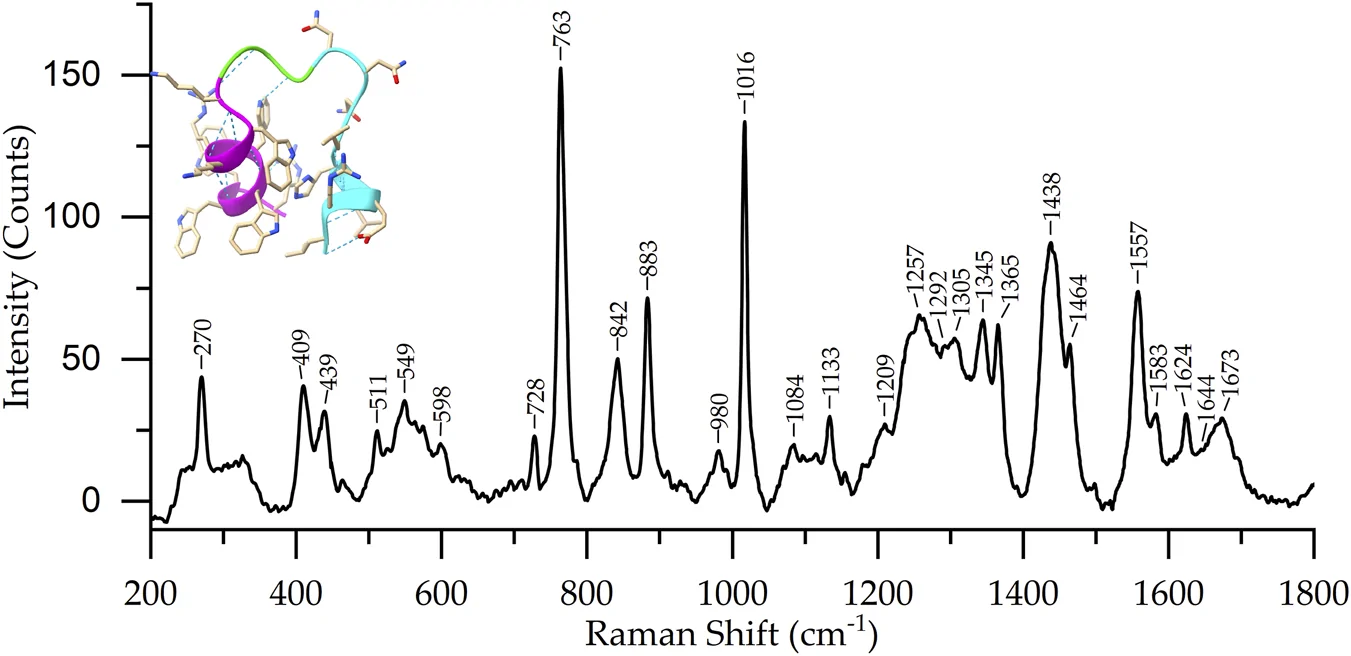
Raman spectrum of COLTAMP. Inset provides the predicted multifunctional peptide structure.

The multifunctional peptide contains multiple repeated amino acid groups in its structure, including four tryptophans (W), two arginines (R), one histidine (H), one leucine (L), and one lysine (K). The four Trp residues dominated the spectra, which was expected because the indole ring is one of the most FTIR- and Raman-active biomolecules. The FTIR spectrum of the multifunctional peptide in the solid state revealed several diagnostic vibrational features characteristic of an organized, hydrogen-bonded peptide backbone. The most structurally informative feature is the amide I band, observed at 1,658 cm^−1^, which arises predominantly from the C=O stretching of the peptide backbone. This position falls within the established α-helical assignment range of 1,647–1,658 cm^−1^, indicating that the α-helical secondary structure is the predominant conformation adopted by the peptide. Importantly, no strong absorption was detected near 1,630 cm^−1^, a region characteristically associated with β-sheet hydrogen bonding patterns, which further argues against a predominantly β-sheet arrangement (Ganim et al., 2008; Tatulian, 2013).

Complementing the amide I signal, the amide II band at 1,533 cm^−1^, originating from the coupled N-H in-plane bending and C-N stretching modes, is consistent with a well-organized peptide backbone (Tatulian, 2013). The amide A band at 3,286 cm^−1^, attributable to N-H stretching, provides additional structural evidence, i.e., its position substantially below the free N-H stretching region (∼3,400–3,450 cm^−1^) is diagnostic of significant intramolecular or intermolecular hydrogen bonding in the solid state (Tatulian, 2013), which is consistent with a stabilized helical geometry.

Additional bands in the fingerprint region at 1,201 and 1,134 cm^−1^ are assigned to the skeletal C-N and C-C stretching vibrations of the peptide backbone. Tryptophan-specific vibrational features were also identified: the band at 747 cm^−1^ corresponds to the indole ring breathing/bending mode, a well-established marker for tryptophan residues, while the band at 722 cm^−1^ arises from the indole ring out-of-plane deformation. The bands at 837 and 801 cm^−1^ are attributed to C-C stretching and aromatic out-of-plane deformations associated with the indole system (Barth, 2000; Andrushchenko et al., 2006).

The Raman spectrum of the multifunctional peptide (Figure 4) is dominated by vibrational signatures from the four tryptophan (W) residues present in its sequence, which is consistent with the well-established high Raman scattering cross-section of the indole chromophore in biomolecular systems. Two highly prominent peaks were observed at 763 and 1,016 cm^−1^. The band at 763 cm^−1^ arises from the in-phase ring breathing mode of the indole ring, whereas that at 1,016 cm^−1^ reflects the out-of-phase ring breathing mode of the tryptophan residues (Wen and Thomas, 2000; Tuma, 2005; Yamashita et al., 2022). Additional tryptophan-associated features include a band at 883 cm^−1^ originating from indole N-H bending vibrations and peaks at 1,557, 1,464, and 1,438 cm^−1^, corresponding to the stretching and bending modes of the indole ring system. The peaks at 1,365 and 1,345 cm^−1^ constitute the Trp Fermi resonance doublet, which is sensitive to both the local environment of the tryptophan residues and the hydrogen-bonding state of the indole N-H group. The band at 842 cm^−1^ is associated with aliphatic side chain contributions and the skeletal framework of the peptide backbone (Wen and Thomas, 2000; Tuma, 2005; Dieng and Schelvis, 2010; Yamashita et al., 2022).

Analysis of the amide I region from a secondary structure perspective revealed a heterogeneous conformational profile in the solid state. The band at 1,644 cm^−1^ supports the presence of α-helical secondary structure, while the moderate peak at 1,673 cm^−1^ is indicative of minor β-turn or antiparallel β-sheet arrangements. In the amide III region, the signal at 1,292 cm^−1^ falls within the range assigned to both α-helical and β-turn conformations, whereas the band at 1,257 cm^−1^ is characteristic of unordered or random coil regions (Williams, 1986; Maiti et al., 2004; Huang et al., 2006). Notably, no strong, sharp band was observed in the 1,230–1,240 cm^−1^ region, which would be diagnostic of an extended β-sheet arrangement, confirming that the β-sheet structure is not a predominant feature of the peptide.

The results from FTIR and Raman spectroscopy aligned with the secondary structure predictions generated by PEP-FOLD 3.5, confirming that COLTAMP predominantly adopts an α-helical conformation, with additional elements such as turns that contribute to its peptide flexibility. These structural insights support the design rationale of the COLTAMP, in which maintaining an α-helical structure is essential for its dual functionality.

### 3.3 Bacterial growth inhibition studies

We conducted a zone of inhibition assay on agar plates to evaluate the antimicrobial efficacy of the COLTAMP and its individual domains against *S. mutans*. Chlorhexidine (CHX) was used as a positive control, and AMP was used as a positive peptide control to screen for peptide activity. CBP was used as a negative peptide control, as no bactericidal activity against *S. mutans* was anticipated. The images were captured using a macro lens attached to a smartphone camera, and the inhibition zones produced by these agents are shown in Figure 5, with quantitative data summarized in the accompanying table (Table 2).

**FIGURE 5:**
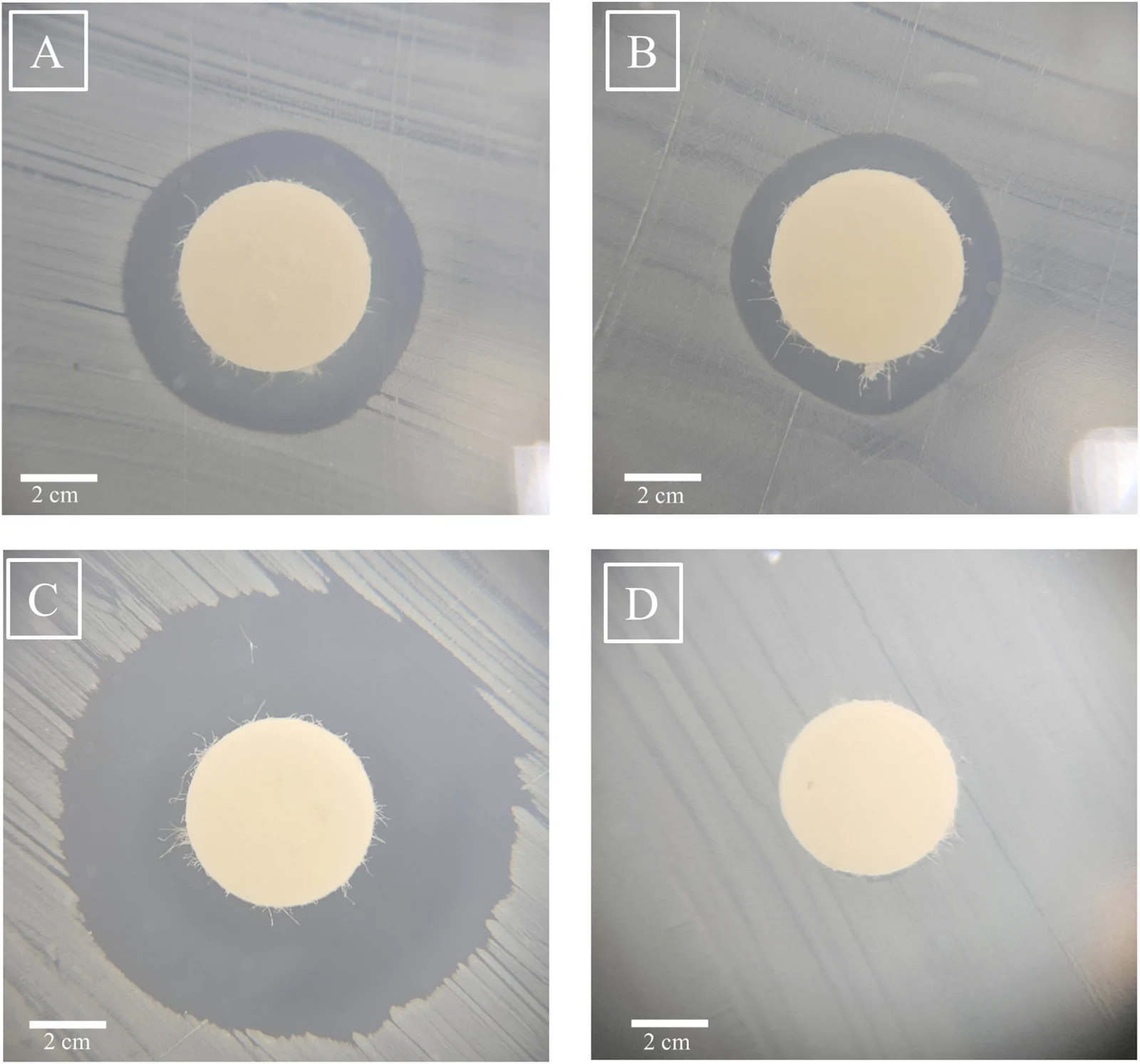
Zone of Inhibition results for AMP, CBP, COLTAMP, and CHX. **(A)** AMP, **(B)** COLTAMP, **(C)** CHX, and **(D)** CBP-treated agars.

**TABLE 2:** Zone of inhibition measurements of the peptides and CHX.

Agent	Sequence	Concentration (mg/mL)	Inhibition zone (mm)
CHX	n/a	0.063	14.67 ± 0.45
AMP	KWKRWWWWR-NH_2_	4.00	9.34 ± 0.29
COLTAMP	LRELHLNNNGGGKWKRWWWWR-NH_2_	4.00	8.44 ± 0.11
CBP	LRELHLNNN	4.00	0

AMP exhibited a zone of inhibition of 9.34 ± 0.29 mm in diameter, indicating its activity against *S. mutans*. It has a smaller inhibition than CHX, a well-established antimicrobial agent commonly used in dental practice due to its potent broad-spectrum activity against oral pathogens, but it still demonstrated effective growth inhibition against *S. mutans*. CBP did not demonstrate any zone of inhibition, indicating that CBP alone does not have antimicrobial properties. In contrast, COLTAMP exhibited a zone of inhibition of 8.44 ± 0.11 mm. The results showed that COLTAMP preserved its antimicrobial properties and demonstrated an effective zone of inhibition.

The bacterial growth inhibition by the COLTAMP peptide against S. mutans was also evaluated in solution using the Resazurin assay. The antimicrobial activity of the COLTAMP was compared to the collagen binding and antimicrobial peptides in addition to chlorhexidine (CHX), which is commonly used antiseptic and disinfectant in dental care, BHI broth and diH2O. The Resazurin assay is a useful tool to measure bacterial activity by utilizing resazurin salt, a blue non-fluorescent dye, which is converted to a pink, fluorescent molecule called resorufin by the metabolic activity of bacteria. The more bacteria present and actively growing, the stronger the fluorescence, indicating a higher activity (Vieira-da-Silva and Castanho, 2023). Bacterial viability was quantified using spectral fluorescence readings at Ex565/Em595. The Supplementary Figure S1 displays in solution activity of the peptides. The COLTAMP showed bacterial growth inhibition starting at 8 μg/mL and fully inhibited the growth (MIC) at 64 μg/mL, while the collagen-binding peptide showed no antimicrobial activity against *S. mutans*.

### 3.4 Morphology studies on *Streptococcus mutans* bacterial membrane via SEM

The effects of AMP and antimicrobial COLTAMP treatment on *S. mutans* were determined using scanning electron microscopy (SEM). Overnight *S. mutans* culture at the mid-logarithmic phase was diluted and then incubated with 4× MIC of AMP and COLTAMP. Untreated bacteria were used as the control. After overnight incubation with the peptide, the bacteria were harvested and processed for SEM imaging. Figure 6 shows the morphological changes in bacterial membranes after incubation with the peptides. In the untreated control group, *S. mutans* appeared as intact, rod-shaped cells with smooth, round surfaces and tightly packed clusters of cells. In contrast, the cells treated with AMP showed clear signs of membrane disruption. The bacterial surfaces appeared irregular, with visible roughness and structural collapse. Similarly, COLTAMP treatment caused pronounced morphological changes. The bacteria in this group showed severe deformation, with many cells appearing swollen or ruptured. This disrupted morphology suggests that the COLTAMP not only compromises membrane integrity but also induces physical damage to the bacterial structure.

**FIGURE 6:**
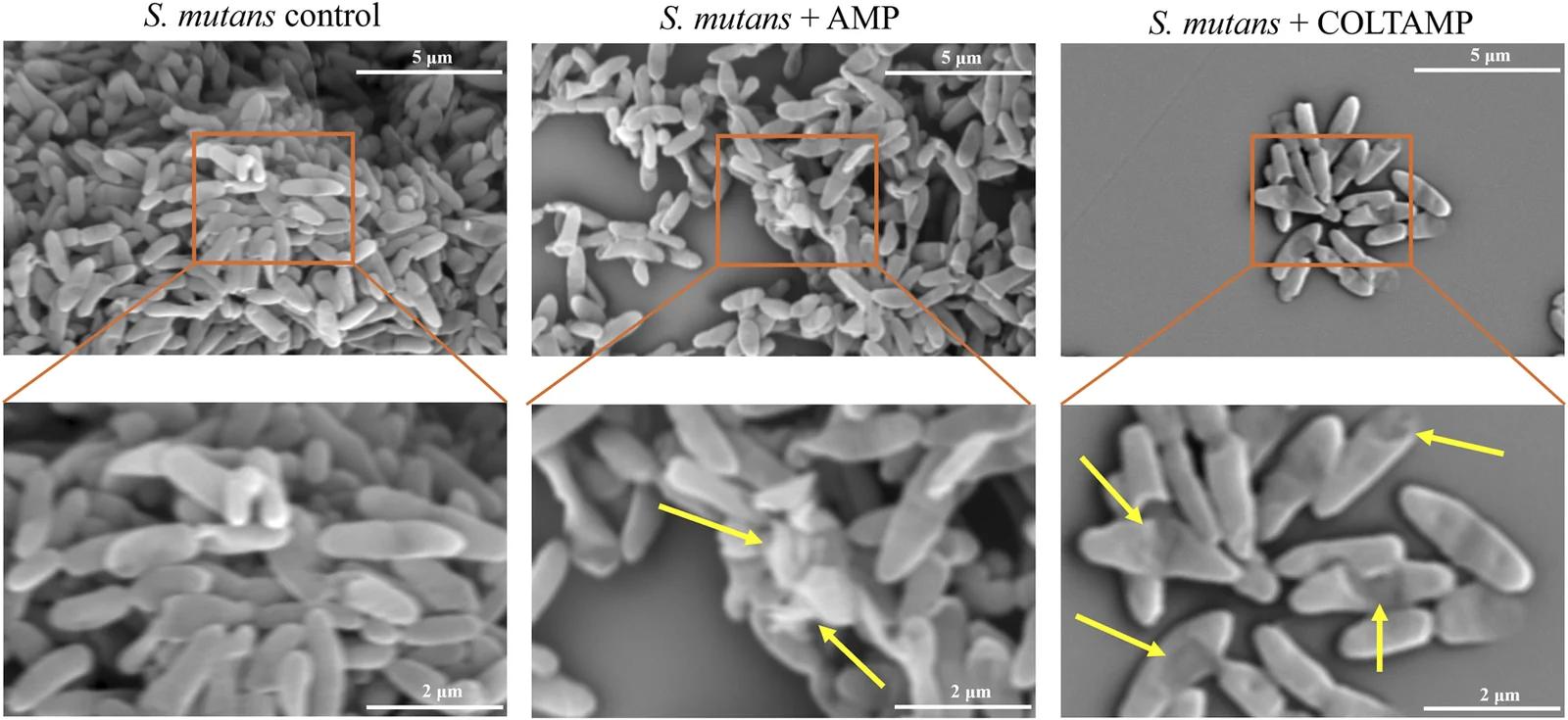
Morphological study on *Streptococcus mutans* after being treated with AMP and COLTAMP via SEM imaging at different magnification levels. Yellow arrows in the middle and the right panel highlight key features of bacterial cell wall disruption, including surface blebbing, wall collapse, and structural fragmentation following peptide exposure.

The morphological changes observed in *S. mutans* after peptide treatment highlight the antimicrobial efficacy of both the AMP and COLTAMP. The intact and smooth bacterial surfaces in the control group contrasted sharply with the disrupted and irregular morphologies observed in the treatment groups, indicating that both AMP and COLTAMP can compromise bacterial membrane integrity.

### 3.5 Collagen fibril assembly and roughness analysis via AFM

The effects of peptide incorporation into collagen on collagen self-assembly were investigated using atomic force microscopy (AFM) topography. Collagen was drop-cast onto clear mica discs with or without peptide incorporation. The samples were left undisturbed overnight (∼16 h) at 4 °C until their surfaces were visibly dry. The images of the collagen samples acquired in tapping mode were analyzed using NanoScope Analysis v3.0 software. They are presented as 5 μm × 5 μm images with a pseudo-color scale next to each image. Non-fibrillar regions, identified as intensity-saturated areas on the surface, correspond to acetic acid salt crystals resulting from the evaporation of the collagen suspension. These areas were excluded from the analysis to prevent signal interference and ensure an accurate characterization of the collagen fibril network. The samples included collagen without any peptides, collagen with CBP, collagen with AMP, and collagen with COLTAMP. The fibril assembly of collagen is presented in Figure 7, and the mean fibril width and roughness analysis are summarized in Table 3.

**FIGURE 7:**
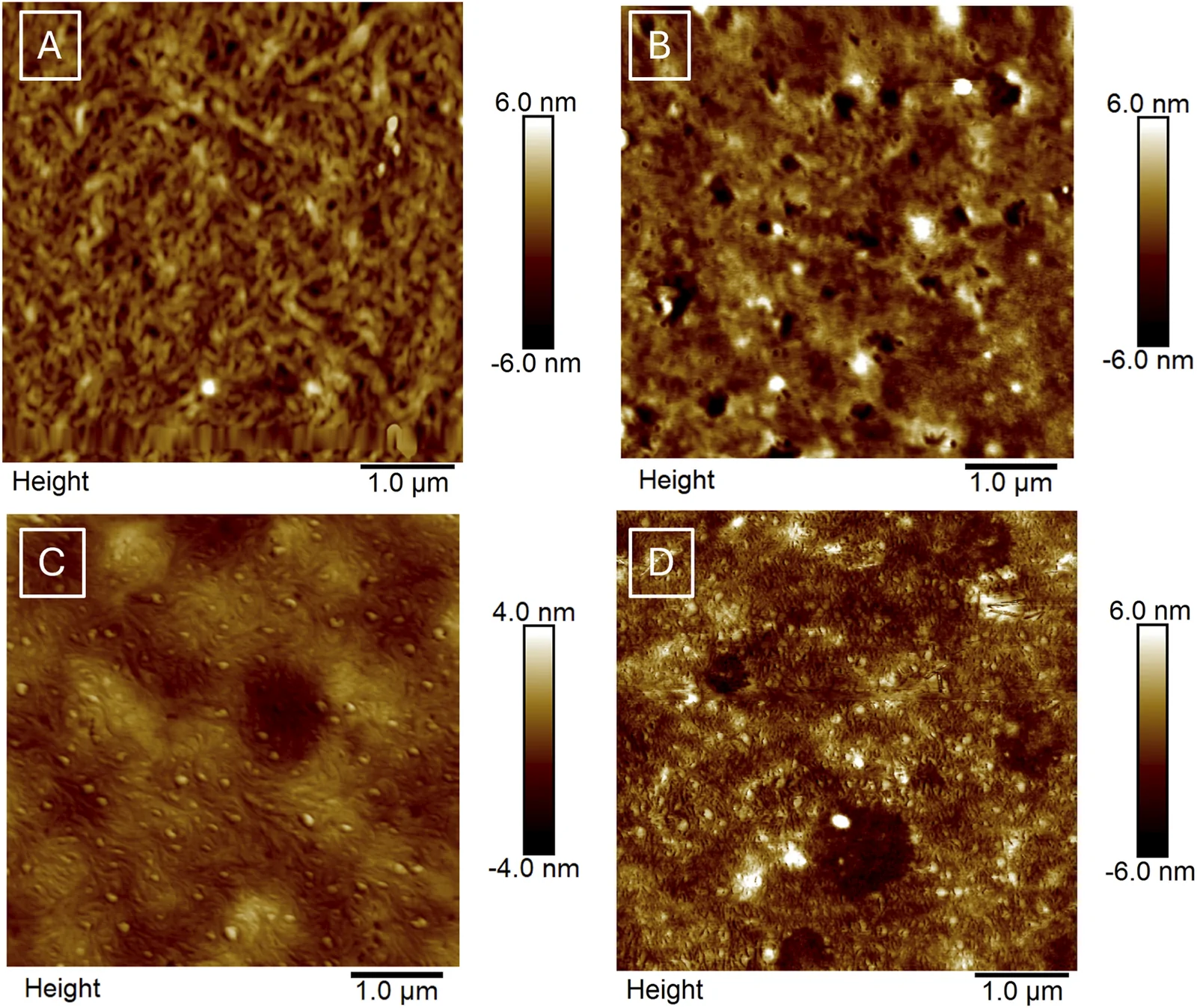
Fibril assembly of collagen with and without peptides. **(A)** Collagen, **(B)** Collagen + CBP, **(C)** Collagen + AMP, and **(D)** Collagen + COLTAMP.

**TABLE 3:** Collagen fibril width and roughness analysis of the samples.

​ Samples	Fibril width			Roughness (nm)		
	Mean fibril width (nm)	Standard deviation	Coefficient of variations (CV)	Rq	Ra	Rmax
Collagen	20.70	1.14	0.055	1.21	0.939	11.5
CBP	24.06	2.19	0.091	1.54	1.18	16.6
AMP	23.20	3.66	0.158	0.602	0.474	5.49
COLTAMP	17.26	2.83	0.164	1.62	1.24	24.7

As shown in Figure 7A (collagen alone), collagen fibrils formed a dense, randomly oriented fibril alignment. The fibrils appeared tightly packed with uniform thickness, consistent with the measured mean fibril width of 20.7 ± 1.14 nm and a low coefficient of variation (CV) of 0.055, indicating a uniform structure. The surface was smooth (Rq: 1.21 nm, Ra: 0.939) with minimal interruptions in the fibrillar networks. The addition of CBP resulted in noticeable changes in the fibrillar structure (Figure 7B). The fibrils appeared thicker and less densely packed, with larger aggregated regions visible across the surface. The mean fibril width increased to 24.06 ± 2.19 nm, with a CV of 0.091, suggesting that CBP facilitates the lateral bundling or aggregation of collagen fibrils. The increase in surface roughness (Rq: 1.54 nm, Ra: 1.18 nm) correlates with the observed larger fibril aggregates and thicker fibrils, which collectively contribute to a more pronounced surface topography. The incorporation of AMP disrupted the typical collagen fibrillar structure (Figure 7C). The fibrils were less distinct and appeared less organized than those of collagen alone or collagen-CBP-treated groups. The mean fibril width of 23.2 ± 3.66 nm and a higher CV of 0.158 indicate reduced uniformity and a broader distribution of fibril widths. The surface became noticeably smoother, as reflected by the reduced roughness values (Rq = 0.602 and Ra = 0.474 nm). This smoother surface and broader fibril distribution suggest that AMP interferes with the hierarchical assembly of collagen fibrils, potentially influencing lateral interactions and leading to flatter assemblies. The COLTAMP (Figure 7D), which combines the effects of AMP and CBP through its dual-domain design, exhibited unique effects on collagen assembly. The fibrils were the narrowest among the samples, with a mean width of 17.26 ± 2.83 nm and the highest CV of 0.164, reflecting both a reduction in fibril size and increased variability in size. The roughness values (Rq: 1.62 nm, Ra: 1.24 nm) were the highest among all the samples, indicating the presence of more pronounced topographical features. The combined effect of the AMP and CBP domains likely creates a balance between promoting localized aggregation and affecting regular fibril assembly, leading to the observed structural and surface changes, combining the individual effects of active domains in the multifunctional design.

The incorporation of peptides altered collagen assembly compared to the controls, as evidenced by changes in fibril width and surface roughness. The collagen-binding peptide (CBP) promoted fibril thickening and increased surface roughness, consistent with its role in enhancing collagen molecule interactions. In contrast, the antimicrobial peptide (AMP) disrupted collagen fibril assembly, leading to thinner fibrils and a smoother surface, likely due to its interaction with collagen molecules that interfere with normal fibril formation. COLTAMP had the most pronounced effects on collagen assembly, resulting in thinner fibrils, suggesting that its dual functionality introduces competing interactions that modulate the typical hierarchical organization of collagen fibrils.

## 4 Discussion

This study demonstrates the potential and versatility of COLTAMP as a multifunctional agent for modulating collagen structure and providing antimicrobial activity. Through a combination of structural analyses, antimicrobial assays, and collagen platform evaluations, we elucidated how the design of the peptide, which combines collagen-binding and antimicrobial domains, affects its functionality in various environments. These findings establish the versatility of the COLTAMP as a bio-inspired material capable of addressing challenges in collagen-based systems while offering avenues for broad applications related to bone and dentin repair.

Taken together, the FTIR and Raman spectroscopic data provide a consistent and detailed picture of the secondary structure of the multifunctional peptide in the solid state. The analysis established that COLTAMP predominantly adopts an α -helical conformation, thereby validating the computational predictions. Characteristic amide band I and amide band III bands were observed in the Raman spectrum, indicating the coexistence of minor β-turn, antiparallel β-sheet, and disordered conformational elements alongside the dominant helical structure. This structural heterogeneity is not unexpected, given the multifunctional nature of the peptide. The leucine-rich repeat domain is intrinsically predisposed to stabilize helical conformations through hydrophobic packing, whereas the tryptophan- and arginine-rich antimicrobial domain adopts more flexible, β-type, or turn-enriched arrangements under solid-state conditions. Therefore, the observed mixture of secondary structural elements is a direct reflection of the domain-level architecture encoded in the peptide sequence. Furthermore, FTIR amide A and amide II vibrations confirmed that the helical segments were stabilized by extensive hydrogen bonding, which is a prerequisite for functional integrity in both collagen-binding and antimicrobial domains. Tryptophan spectral signatures in the Raman spectrum reveal environmental heterogeneity among the indole side chains distributed across structurally distinct domains.

These findings reinforce the structural logic underlying the design of multifunctional peptides. Maintaining an α-helical backbone is essential for the amphipathic character of the peptide, which enables the spatial segregation of hydrophobic and hydrophilic residues required for both membrane interactions and collagen fibril binding. The coexistence of turns and disordered elements likely confers conformational flexibility at the domain junction, which may facilitate the independent function of each domain in distinct biological environments. Collectively, the convergence of computational predictions and experimental spectroscopic validation confirmed that the multifunctional peptide retained the structural features necessary to support its intended dual functionality.

The antimicrobial activity of the COLTAMP was evaluated using the zone of inhibition assay. The results demonstrated that the COLTAMP maintained substantial activity with a distinct inhibition zone that remained close to that of AMP alone. These findings indicate that the incorporation of the collagen-binding domain did not disrupt the antimicrobial function of the peptide, as the multifunctional design produced a clear inhibition zone against *S. mutans*, whereas CBP alone showed no detectable activity. The absence of inhibition in the CBP group supports its role as a non-bactericidal targeting domain, whereas the AMP control confirmed that the antimicrobial domain was responsible for the observed bacterial growth inhibition. Although COLTAMP produced a modestly smaller inhibition zone than AMP alone, this trade-off is consistent with its intended function as a dual-domain agent rather than a limitation of its design. The addition of the collagen-binding domain confers targeted, self-anchoring localization to collagen substrates, a property AMP alone does not possess. This localized retention at the site of application may be more relevant to therapeutic performance than the size of the inhibition zone in a diffusion-based assay, which does not capture anchoring or sustained local activity at a tissue interface.

While the zone of inhibition assay demonstrated the bactericidal efficacy of the COLTAMP, SEM analysis provided complementary insights into the mechanism of bacterial inhibition. Intact rod-shaped *S. mutans* cells in the control group provided a baseline for the healthy bacterial morphology. In contrast, AMP-treated bacteria exhibited characteristic signs of bacterial cell wall disruption, including surface roughness and blebbing, indicative of pore formation and leakage of intracellular content in the bacterial cells. The COLTAMP-treated group displayed more pronounced morphological alterations, including substantial cell wall collapse, irregular deformation, and extensive structural fragmentation. This suggests that COLTAMP not only retains the cell wall-disruptive properties of the AMP domain but may also introduce additional destabilizing interactions owing to its dual-domain architecture. These findings reinforce the hypothesis that the COLTAMP acts through a combination of direct cell wall disruption and potential interference with bacterial cell wall integrity, further validating its antimicrobial potential. The structural modifications observed in *S. mutans* cells following COLTAMP treatment provided critical visual evidence of its mechanism of action at the cellular level, highlighting the peptide’s adaptability and ability to function effectively in a multifunctional design.

AFM imaging revealed the effects of peptide incorporation on the hierarchical assembly and structural organization of collagen fibrils. Collagen alone exhibited tightly packed fibrils with uniform widths and smooth surfaces, indicative of organized self-assembly. Analysis of the topography of collagen fibrils suggested that CBP promotes lateral bundling of fibrils, likely through specific interactions that stabilize the intermolecular binding domains. The elevated surface roughness further supports the notion that CBP enhances fibril aggregation. In contrast, the incorporation of AMP led to alterations in the fibrillar organization of collagen. The amphipathic nature of AMP may facilitate interactions with both hydrophilic and hydrophobic regions of collagen, altering the electrostatic and steric forces that govern fibril formation. However, COLTAMP induced both a reduction in fibril width and an increase in surface roughness, reflecting its unique ability to modulate the hierarchical organization of collagen. Quantitative analysis suggests that COLTAMP limits lateral aggregation while introducing localized modulations in fibril packing. The multifunctional design of COLTAMP suggests that CBP-driven stabilization and AMP-induced modulation occur simultaneously, leading to an altered fibrillar architecture. The collagen-binding domain likely promotes localized aggregation, stabilizing certain regions of the fibrillar network, whereas the antimicrobial domain interferes with the regular packing of collagen molecules, leading to thinner fibrils. The ability to modulate fibril aggregation, width, and roughness through peptide design provides a strategic approach for engineering collagen-based biomaterials with optimized structural and functional properties. Additionally, the differential effects of CBP, AMP, and COLTAMP underscore the importance of fine-tuning peptide-collagen interactions to achieve the desired material properties.

Collectively, these findings position the COLTAMP as a promising candidate for antimicrobial collagen-based materials. While this dual functionality of the COLTAMP offers a targeted antimicrobial delivery to collagen-based scaffolds or heterogenous tissue such as bone and dentin, future studies should focus on evaluating its cellular safety profiling and biocompatibility analysis for clinical translation. Liquid-state conformation and quantitative antimicrobial metrics on diverse bacterial species would further explore the translational potential of COLTAMP for biomedical applications as a promising approach.

Collagen-based biomaterials have many biomedical applications, including drug delivery, hemostatic agents, skin and soft tissue repair, tendon repair, bone repair, and periodontal regeneration. The benefits of COLTAMP would be most appreciated in potentially non-sterile environments where bacterial contamination is more likely, ultimately compromising repair. This includes the oral cavity and clinical situations in which soft tissues covering the repairable tissues of concern are compromised owing to issues such as mechanical trauma, vascular injury, cancer, radiation therapy, or scarring from prior surgical procedures. Immunocompromised hosts are also at an increased risk. Clinical examples would include open fracture repair, burn wounds, Achilles tendon repair, craniotomy in the setting of tumor resection, mandibular reconstruction in the setting of head and neck cancer ± radiation trauma, inferior orbital fractures, and bone cancer resection in the setting of radiation trauma (Rezvani Ghomi et al., 2021; Fan et al., 2023; Li et al., 2025).

Such materials can be employed in wound dressings and tissue engineering scaffolds and utilized as bioactive coatings that display different agents for medical and dental implants. The ability to modulate collagen assembly while maintaining antimicrobial activity offers a unique advantage in addressing the challenges in which bacterial colonization and structural integrity are critical.

## 5 Conclusion

Collagen is the most abundant protein in the body across diverse tissues, including bone, oral cavity, skin, cartilage, tendon, and cornea among others. Consequently, collagen-based scaffolds have demonstrated great potential in facilitating tissue repair, reconstruction and regeneration across these tissues. However, any rupture among these tissues, a wound, an implant insertion, a periodontal pocket, or a fracture exposes collagen to pathogen attack. Its dense structure also provides shielding for pathogens bound to collagen, where antibiotics and/or antimicrobial agents may have limited penetration. We designed a multifunctional peptide, COLTAMP, incorporating an antimicrobial peptide with collagen-binding properties. COLTAMP was evaluated with respect to its antimicrobial and collagen self-assembly properties. FTIR and Raman spectroscopy confirmed that the multifunctional peptide adopts a predominantly α-helical conformation stabilized by extensive hydrogen bonding, with minor β-turn and disordered elements reflecting the structural heterogeneity inherent to its multifunctional domain architecture. The peptide structure was investigated to confirm its potential interactions with collagen and bacterial membranes, and the peptide was shown to be active against *S. mutans* using a zone-of-inhibition assay. SEM studies provided insights into the mechanism of action and revealed that the COLTAMP caused bacterial cell wall disruption. Compared to AMP, COLTAMP demonstrated pronounced effects on cell wall disruption, suggesting that the collagen-targeted activity of COLTAMP may contribute to its spatial distribution across collagen fibrils, resulting in enhanced antimicrobial activity. Our AFM analyses revealed that COLTAMP incorporation modulated collagen self-assembly by introducing heterogeneity in fibril width and surface topography. Its dual-domain architecture, linked by a flexible spacer, likely introduced competing interactions that disrupted the typical assembly dynamics of collagen, creating thinner, irregular fibrils with high surface roughness. The multifunctionality of COLTAMP offers a peptide-based tunable approach to provide microbial inhibition and collagen-binding in scaffolds used in tissue repair, reconstruction, and regeneration.

## Data Availability

The original contributions presented in the study are included in the article/Supplementary Material, further inquiries can be directed to the corresponding author.
