# Supplementary material for: Collagen targeted self-anchoring antimicrobial molecular agents
Source: Front Bioeng Biotechnol. 2026 Oct 2;14:1914224. doi: 10.3389/fbioe.2026.1914224 (PMC13672547; doi:10.3389/fbioe.2026.1914224)
Supplement: Supplementary file 1 [file DataSheet1.pdf]

Collagen Targeted Self-Anchoring Antimicrobial Molecular Agents

Nur Ceren Selcuk [1,2], Erhan Demirel [2], Stephen C. Waller [3], Malcolm L. Snead [2,4], Paulette Spencer [1,2,5], Candan Tamerler [1,2,5]*

[1]Bioengineering Program, University of Kansas, Lawrence, KS, United States

[2]Institute for Bioengineering Research, University of Kansas, Lawrence, KS, United States

[3]Departments of Microbiology, Molecular Genetics & Immunology and Infectious Diseases, University of Kansas Medical Center, Kansas City, KS, United States

[4]Center for Craniofacial Molecular Biology, Herman Ostrow School of Dentistry of USC, The University of Southern California, Los Angeles, CA, United States

[5]Department of Mechanical Engineering, University of Kansas, Lawrence, KS, United States

*** Correspondence:**
Candan Tamerler
ctamerler@ku.edu

1 Bacterial Growth Inhibition of ColTAMP against *S. mutans* with Resazurin Assay

Antimicrobial activity of ColTAMP against *S. mutans* was evaluated in solution with standard broth microdilution assay and bacterial growth inhibition analyzed by Resazurin assay. In this assay, $1x10^4$ CFU/mL bacteria were incubated with ColTAMP peptide at concentrations ranging from 1 to 64 µg/mL at 37 ºC in the presence of 5% CO_2 overnight and bacteria viability was assessed with Resazurin assay. The antimicrobial peptide (AMP) and collagen-binding peptide (CBP) were tested alongside ColTAMP as positive and negative peptide controls, respectively. CHX was included as an antimicrobial positive control, bacteria incubated without antimicrobial treatment served as the untreated growth control, and wells containing BHI broth and DI H_2O were used as blank controls. The Resazurin assay measures bacterial activity by utilizing resazurin salt, a blue non-fluorescent dye, which is reduced by metabolically active bacteria to a pink, fluorescent product resorufin, allowing bacterial metabolic activity to be quantified relative to the untreated control (Vieira-da-Silva and Castanho, 2023). The more bacteria present and actively growing, the stronger the fluorescence, indicating higher activity.

A 20X resazurin stock solution was prepared by dissolving resazurin salt (Sigma-Aldrich 199303) in sterile 1X PBS at 200 µg/mL, followed by sterile filtration through a 0.22 µm filter while protecting the solution from light. The stock solution was aliquoted into sterile containers and stored at −20 °C to minimize repeated freeze-thaw cycles. Prior to use, the 20X stock was thawed and diluted in assay medium or buffer to a 1X working concentration for addition to the 96-well plate.

Following overnight incubation, bacterial cultures were transferred to a sterile 96-well plate containing fresh BHI medium, and 1X resazurin solution was added at 10% (v/v). After 2h incubation,

fluorescence was measured at Ex/Em = 565/595 nm. The resulting antimicrobial activity are shown in Figure S1.

The AMP showed strong in-solution antimicrobial activity, reaching its MIC at 8 µg/mL whereas the CBP showed no detectable antimicrobial activity over the tested concentration range. Incorporation of AMP into the ColTAMP preserved its antimicrobial function against *S. mutans*. ColTAMP showed inhibition of bacterial metabolic activity beginning at 8 µg/mL, with minimum inhibitory concentration of 64 µg/mL. These results demonstrated that the antimicrobial domain remained functionally active following incorporation into the multifunctional peptide. Consistent with its intended role as the targeting domain, the lack of activity of the CBP further indicates that the antimicrobial response observed for ColTAMP is associated with the incorporated AMP domain rather than the collagen-binding sequence alone.

1.1 Supplementary Figures

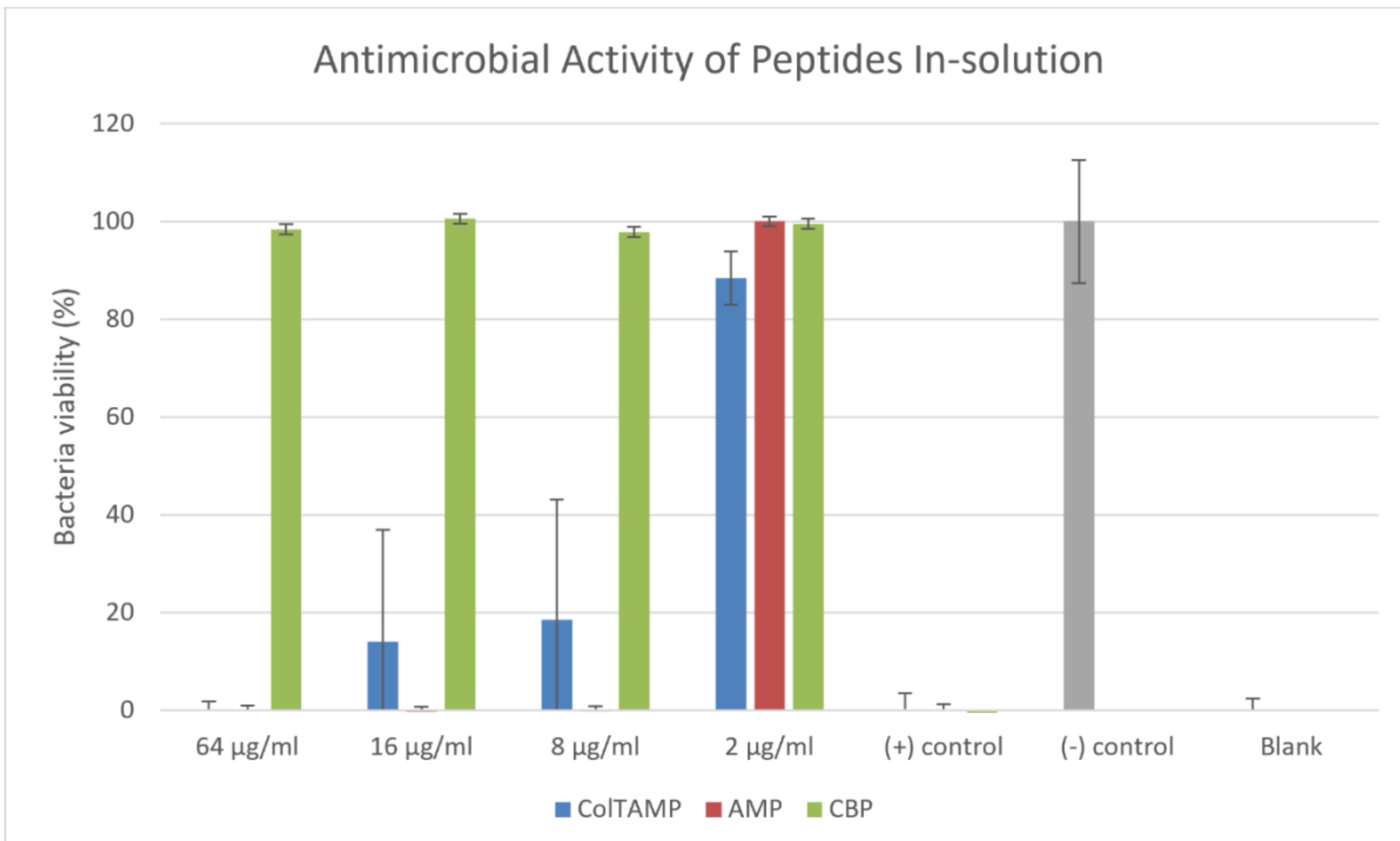

Supplementary Figure 1. Antimicrobial activity of ColTAMP against *S. mutans* with Resazurin assay in solution. Dark blue bars represent ColTAMP activity results, red bars represent AMP results, and green bars represent collagen binding peptide activity.

2 References

Vieira-da-Silva, B., and Castanho, M. (2023). Resazurin Reduction-Based Assays Revisited: Guidelines for Accurate Reporting of Relative Differences on Metabolic Status. *Molecules* 28(5). doi: 10.3390/molecules28052283.
